# Biomarkers of exposure to polycyclic aromatic hydrocarbons in urine of municipal police officers: impact of inhalation on total exposure

**DOI:** 10.1007/s11356-025-36342-2

**Published:** 2025-04-08

**Authors:** Veronika Gomersall, Katerina Ciglova, Ondrej Parizek, Andrea Rössnerova, Pavel Rössner, Radim J. Sram, Jan Topinka, Jana Pulkrabova

**Affiliations:** 1 Faculty of Food and Biochemical Technology, Department of Food Analysis and Nutrition, University of Chemistry and Technology, Prague, Technicka 3, 166 28 Prague 6, Czech Republic; 2 Department of Toxicology and Molecular Epidemiology, Institute of Experimental Medicine CAS, Videnska 1083, 142 20 Prague 4, Czech Republic; 3 Department of Genetic Toxicology and Epigenetics, Institute of Experimental Medicine CAS, Videnska 1083, 142 20 Prague 4, Czech Republic

**Keywords:** Air pollution, Biomonitoring, Czech Republic, GC–MS/MS, Personal air samplers, UHPLC − MS/MS, Urine analysis, PAHs

## Abstract

Polycyclic aromatic hydrocarbons (PAHs) are ubiquitous environmental contaminants that adversely affect human health, mainly through their carcinogenic and mutagenic properties. Monitoring the exposure to PAHs of the inhabitants of air polluted regions is important because of the impact of these pollutants on human health. The aim of this study was to assess the exposure to PAHs of municipal police officers (non-smokers) living in three localities in the Czech Republic (strategically selected according to the level of air pollution) and determine how air pollution impacts personal exposure to PAHs via inhalation. Twenty PAHs were determined in the inhaled air collected from personal air samplers. Simultaneously, the total exposure to PAHs was investigated by evaluating the concentrations of 11 monohydroxylated polycyclic aromatic hydrocarbons (OH-PAHs) in urine samples. Despite the observed differences in the concentrations of PAHs in the air from the personal samplers between the three locations, no statistically significant differences were found in the concentrations of OH-PAHs in the urine samples. Outcomes of this study indicate that inhalation exposure is not the primary source of PAHs exposure for Czech municipal police officers, but that diet may also be an important contributor to total body burden. The levels of OH-PAHs found in urine of Czech municipal police officers were very similar to those found in the urine of the Czech mothers from our previous study. This study provided the data about the body burden of potentially occupationally exposed group that has not yet been studied in the Czech Republic.

**Graphical abstract:**

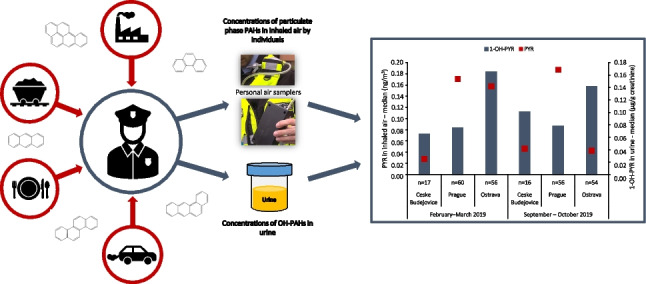

**Supplementary Information:**

The online version contains supplementary material available at 10.1007/s11356-025-36342-2.

## Introduction

Polycyclic aromatic hydrocarbons (PAHs) are well-known and studied environmental contaminants that are formed during the pyrolysis and pyrosynthesis of organic matter and indicate the level of air pollution from anthropogenic sources (such as heavy industry, exhaust fumes or local heating systems) and non-anthropogenic sources (such as volcanic activity or forest fires) (Purcaro et al. 2013; Abdel-Shafy and Mansour 2016). These contaminants are also formed in food during culinary practices such as grilling or smoking. They can also enter food from a contaminated environment, by sorption from soil, or by deposition of particulate matter (PM) from the air (EFSA 2008).

Human exposure to these substances is associated with adverse human health effects. Of most significant concern is the carcinogenicity of some of the PAHs’ reactive metabolites, e.g. benzo(*a*)pyrene (BaP), which, due to its reactive metabolites, is included in the database of the International Agency for Research on Cancer (IARC) as carcinogenic to humans (Group 1) (IARC 2021). Higher exposure to PAHs has been associated with an increased risk of lung, skin or gastrointestinal cancers (Sun et al. 2021; Ravanbakhsh et al. 2023). Additionally, reactive metabolites of some PAHs show toxicity, mutagenicity and teratogenicity and can also act as endocrine disruptors. Higher human exposure to PAHs is also linked with a higher incidence of childhood obesity (Poursafa et al. 2018; Bushnik et al. 2020), respiratory problems (Sram et al. 2013) and male infertility (Xia et al. 2009; Yang et al. 2017)*.*

Given the different sources of PAHs, exposure pathways and the load of the body burden are influenced by aspects such as occupation, lifestyle, diet and place of residence. In the case of non-smokers, the diet accounts for 70% (EFSA 2008) to 90% (Polachova et al. 2020) of overall exposure. Another source of PAHs contributing to overall human exposure is polluted air from local sources such as industry, heating by burning wood/coal or traffic (Duan et al. 2014; Li et al. 2010). For occupationally exposed workers, dermal exposure can also be a significant contributor to total exposure along with inhalation (Louro et al. 2022).

The load of the body burden can be assessed by environmental monitoring followed by estimation of daily intake. Dietary exposure to PAHs is investigated by analysing either individual food items or food duplicates of participants’ everyday diets (Polachova et al. 2020). For the estimation of inhalation exposure, analysing the airborne PAHs, sampled by stationary high-volume air samplers (Polachova et al. 2020) or personal air samplers (Duan et al. 2014; Nethery et al. 2012; Perico et al. 2010), is applied. The second technique provides more accurate information about the levels of airborne PAHs to which the person has been actually exposed through the whole sampling period. Another approach to assessing the human body burden of PAHs is human biological monitoring. By measuring biomarkers in human urine, it is possible to determine recent total exposure to PAHs. The primary metabolites excreted in urine are monohydroxalated PAH (OH-PAH) conjugates, usually used as exposure biomarkers (Onyemauwa et al. 2009). Biomonitoring data provides more accurate information for assessing the total human body burden of PAHs, which might be used in public health research to determine what levels of PAHs might be associated with potential adverse effects on human health (Koch et al. 2014; Li et al. 2010).

However, the biomonitoring data provides no information specific to the route. Thus, in studies of particular cohort groups with a potential high risk of exposure via inhalation, the combination of both is beneficial for assessing the origin of human exposure (Li et al. 2010). In this study, we focus on exposure of municipal police offices with residency in three localities of the Czech Republic (air-polluted location, capital city and control group). This potentially occupationally exposed group spent the vast majority of their working hours outdoors and thus been exposed to airborne PAHs from the urban environment contaminated by industry, traffic or local heating. For evaluation on how local air pollution impacts personal exposure of participants to PAHs, the analysis of 11 biomarkers in their one-spot urine and the analysis of 20 airborne PAHs sampled by personal air samplers was conducted. This study also provides a knowledge about the exposure to PAHs of the inhabitants of one of the most air polluted area in the Czech Republic— Ostrava.

## Materials and methods

### Standards, chemicals and other materials

The list of certified standards, chemicals and other materials used and their manufacturers are given in Table S1 and Table S2. For OH-PAHs, calibration solutions (*n* = 13) were prepared in concentrations of 0.01–100 ng/mL in methanol with a concentration of isotopically labelled standards of 10 ng/mL and stored in the freezer (− 20 °C). The Standard Reference Material® (SRM) 3673 applied for the validation experiments was purchased from the NIST (USA). The PAHs working solutions (*n* = 8) were made in 0.05–100 ng/mL concentrations with isotopically labelled standards of 2 ng/mL and stored in a refrigerator (5 °C).

### Sample collection

All samples were provided by the Institute of Experimental Medicine (IEM), Academy of Sciences of the Czech Republic in Prague and the University of Ostrava as part of the molecular-epidemiological study to assess the impact of air pollution on the genome of municipal police officers. This study is part of the Healthy Aging in Industrial Environment (HAIE) project (*HAIE *n.d), which focuses on the effects of specific environmental and lifestyle risk factors on population health and ageing in industrial regions. The Ethics Committee of IEM approved the study. Each participant signed a written consent form and completed a questionnaire about personal information, long-term exposure, diet and short-term exposure. All participants were non-smoking males. Smokers and subjects with medical treatment were not included in the study. Tables 1 and 2 show the key information about them.

**Table 1:** Key information on the municipal police officers

		Ceske Budejovice		Prague		Ostrava	
		*February–March 2019*	*September–October 2019*	*February–March 2019*	*September–October 2019*	*February–March 2019*	*September–October 2019*
Number of participants		17	16	60	56	56	54
Age^a^		38	38	38	39	42	42
		(22–48)	(22–48)	(23–63)	(23–63)	(21–61)	(21–61)
BMI^a^		28	28	29	29	28	28
		(23–41)	(23–41)	(19–37)	(19–37)	(20–45)	(20–45)
Duration of time spent in current job (years)^a^		7.8 (0.8–26.6)		16.0 (0.8–26.8)		11.5 (1.0–25.9)	
Residency in the region of study city (years/age)^a^		1.0 (0.2–1.0)		0.3 (0.0–1.0)		1.0 (0.6–1.0)	
Preferred diet^b^	Plant-based	0%		0%		0%	
	Meat-based	24%		17%		7%	
	Mixed	76%		83%		93%	
Preferred food preparation^b^	Boiling/steaming	18%		30%		32%	
	Frying/barbequing	6%		2%		5%	
	No preference/all type above	76%		65%		66%	
Preferred style of cooking^b^	Gas/electric cooker	100%		100%		98%	
	Stove	0%		0%		2%	

*BMI* body mass index

^a^Median (min–max)

^b^% of participants (from first round)

**Table 2:** Short-term exposure of municipal police officers (first sampling round)

Time spent… (hours)		… at home (inside)	… at work (inside)	TOTAL inside	… at traveling to work	… at home (outside)	… at work (outside)	TOTAL outside
Median (min–max)	Ceske Budejovice	10	3	12	1	2	9	11
		(5–15)	(0–13)	(8–22)	(0–2)	(0–6)	(0–13)	(0–15)
	Praha	11	3	14	1	0	9	9
		(6–20)	(0–12)	(10–23)	(0–5)	(0–6)	(0–12)	(0–12)
	Ostrava	11	2	14	1	0	9	9
		(9–14)	(0–12)	(10–22)	(0–2)	(0–3)	(0–12)	(0–13)

Sampling was conducted in two periods with the same participants, February–March 2019 (1st period) and September–October 2019 (2nd period). Sampling was conducted in three regions of the Czech Republic—Ceske Budejovice (control location), Prague (capital city) and Ostrava (air-polluted location)—which were strategically selected according to the level of air pollution (CHMI 2018). The place with the highest air pollution was Ostrava due to heavy industrial activity in the surrounding area and local heating. Prague was chosen as a densely populated and heavily trafficked capital city, and Ceske Budejovice was selected as a control location.

Ambient air was sampled using personal air samplers—Active PV 1.7 monitors (URG Corp, Chapel Hill, NC, USA) were fit out with Teflon-impregnated glass fibre filters (T60A20, Pallflex) and collected PM2.5 particles (Williams et al. 1999). The volume of air sampled ranged from 2 to 3 m^3^ per sampling period (24 h). The personal air samplers were attached to the officer’s clothing near their faces for 24 h. The urine samples were collected from healthy non-smoking policemen at the end of their shifts into 50-mL tubes (Greiner Bio-one, Kremsmünster, Austria). The 259 urine samples and 259 filters collected were frozen (–20 °C) before analysis.

### Methods

#### Analysis of OH-PAHs in urine samples

Extraction of hydrolysed urine samples by shaking with ethyl acetate followed by a purification step by dispersive solid-phase extraction (d-SPE, with sorbent Z-Sep) is described in detail by Lankova et al. (2016). More detailed information on the analysis can be found in the Supplementary Material. The quantitative and qualitative transitions are shown in Table S3. Creatinine levels were used to normalise urine sample dilution in each sample, and these levels were determined using Jaffe’s spectrophotometric method (Lankova et al. 2016).

#### Analysis of PAHs in filter samples

A filter for air sampling was extracted in a glass test tube with 10 mL of the solvent mixture of dichloromethan:*n*-hexane (1:3, *v/v*) supported by ultrasonic (2 × 30 min). The combined crude extracts were evaporated to near dryness and finished with a gentle stream of nitrogen. The residue was dissolved in 250 µL of an isotopically labelled standard of PAHs (at a concentration of 2 ng/mL) in isooctane. More detailed information on the analysis can be found in the Supplementary Material.

### Quality assurance/quality control and validation

#### OH-PAHs in urine samples

The analytical method for the analysis of 11 OH-PAHs and the spectrophotometric Jaffe’s method for the determination of creatinine concentration were validated using the NIST SRM 3673. For the compounds not certified in the SRM 3673 (chrysene-6-ol (6-OH-CHR) and benzo[*a*]pyrene-3-ol (3-OH-BaP)), the performance characteristics were determined by analysing the artificially contaminated urine blank sample, measured before validation to ensure that 6-OH-CHR and 3-OH-BaP were not naturally present in this sample. For performance characteristics of the UHPLC-MS/MS method, see Table S4.

To compensate for losses during extraction and also for unexpected matrix effects, isotopically labelled analogues were used (in the case of 6-OH-CHR [^2^H]_9_-pyrene-1-ol (d_9_−1-OH-PY)) and in the case phenanthrene-4-ol (4-OH-PHE) [^2^H])_8_-phenanthrene-9-ol (d_8_−9-OH-PHE) were used). Background contamination for all target analytes was controlled by the procedural blank. Sample with 5 mL of deionised water and one sample in two replicates were prepared simultaneously with each batch of samples. The contamination level found in the blank sample was subtracted from each sample prepared in the same batch. The blank samples contained traces of naphthalene-1-ol (1-OH-NAP), naphthalene-2-ol (2-OH-NAP) and pyrene-1-ol (1-OH-PY) (concentrations below 0.03 ng/mL urine). These are common contaminants caused by traces of OH-PAHs in the solvents and ambient air.

#### PAHs in filter samples

The analytical method for analysing 20 PAHs was validated through artificially contaminated unused air sampling filters. For performance characteristics of the GC–MS/MS method, see Table S5.

For the compensation matrix effects, isotopically labelled analogues were used (in the case of benzo[*c*]fluorene (BcFL) ^13^C_3_-pyrene (^13^C_3_-PY), cyclopenta[*c,d*] (CPP) and 5-methylchrysene (5MC) ^13^C_6_-chrysene (^13^C_6_-CHR), benzo[*j*]fluoranthene (BjFA) ^13^C_4_-benzo[*a*]pyrene (^13^C_4_-BaP), dibenzo[*a,l*]pyrene (DBalP) and dibenzo[*a,h*]pyrene (DBahP) ^13^C_12_-dibenzo[*a,i*]pyrene (^13^C_12_-DBaiP) were used). Before the sampling, a non-used filter for air sampling was analysed to check for possible contamination by PAHs. With each batch of samples, background contamination was tested by preparing a procedural blank. The concentration of contamination in the blank sample was subtracted from all samples prepared on the same day. It contained traces of phenanthrene (PHE), fluoranthene (FLA) and pyrene (PY) (average concentrations ranged from 0.05 to 0.4 ng/filter). These are common contaminations caused by traces of PAHs in the solvents and in the ambient air.

### Health risk analysis

Toxicity equivalent concentration (TEQ; given by Eq. 1 and Eq. 2) and inhalation cancer risk (ICR; given by Eq. 3) were used to assessed the human respiratory risk from exposure to PM2.5-bound PAHs. Toxic equivalent factor (TEF) values from the Nisbet and LaGoy (1992) study and the IUR_BaP_ value 8.7 × 10^−5^ ng/m^3^ given by the World Health Organisation (WHO 2000) were used.

1 $$TEQ=\sum i \left[{c}_{i}\times {TEF}_{i}\right]$$

2 $$TEQ=0.001 \left(PHE+FLA+PY\right)+0.01 \left(AN+CHR+BghiP\right)+0.1 \left(BaA+BbF+BkF+IP\right)+BaP+DBahA$$

3 $$ICR=TEQ \times {IUR}_{BaP}$$

### Statistical analysis

The concentrations of the measured 11 OH-PAHs in urine samples are stated in the concentration ratio OH-PAH/creatinine to compensate for differences between the hydration status of each municipal police officer. The results for PAHs in filter samples were calculated as the analyte concentration in the sample (ng/mL of solvent) divided by the total volume of air sampled (m^3^). For results below limit of quantification (LOQ) and with ≥ 50% positive samples (for 259 samples), a value ½ of LOQ was used to calculate the arithmetic mean, median and 5th and 95th percentile.

A logarithmic transformation was performed before each univariate statistical test to provide a normal data distribution. Analysis of variance (one-way ANOVA) followed by Tukey–Kramer’s post hoc test was used to determine statistically significant differences in the concentrations of the monitored compounds between groups of samples varying in geographic location and durations of time outdoors. A paired two-sample *t*-test was applied to evaluate interindividual differences in sampling periods. The Pearson’s correlation coefficient was used to determine correlations between individual exposure to parent compounds and levels of their metabolites in the urine.

## Results

### Concentrations of OH-PAHs in urine

Evaluation of 11 OH-PAHs in the urine of municipal police officers (February–March 2019 (1st period): *n* = 133; and September–October 2019 (2nd period): *n* = 126) living in three locations of the Czech Republic was performed. Summary information on measured concentrations (µg/g creatinine) is presented in Table 3.

**Table 3:** Measured concentrations (µg/g creatinine) of nine OH-PAHs in the urine of municipal police officers sorted by sampling periods

µg/g creatinine	LOQs	February–March 2019 (1st period)—ALL PARTICIPANTS							September–October 2019 (2nd period)—ALL PARTICIPANTS						
		*n* = 133							*n* = 126						
		Positive samples (%)	Arithmetic mean	Median	5th Percentile	95th Percentile	MIN	MAX	Positive samples (%)	Arithmetic mean	Median	5th Percentile	95th Percentile	MIN	MAX
2-OH-NAP	0.001	100	5.17	3.07	1.21	17.7	0.833	27.0	100	5.00	3.99	1.29	10.8	0.738	28.7
1-OH-NAP	0.002	88	0.511	0.309	0.009	1.69	0.0377	3.93	100	1.30	0.530	0.111	4.84	0.0471	21.2
2-OH-FL	0.001	95	0.429	0.312	0.011	1.177	0.0129	2.26	100	0.508	0.332	0.134	1.29	0.100	7.05
2-OH-PHE	0.001	99	0.103	0.072	0.028	0.290	0.0116	0.595	100	0.173	0.120	0.048	0.525	0.0418	0.96
3-OH-PHE	0.001	99	0.141	0.102	0.034	0.399	0.0136	0.901	100	0.161	0.106	0.040	0.422	0.0189	1.36
1-OH-PHE	0.002	100	0.262	0.185	0.070	0.793	0.0455	1.84	100	0.319	0.212	0.082	0.896	0.0439	2.03
9-OH-PHE	0.005	98	0.089	0.059	0.025	0.258	0.0170	0.517	99	0.155	0.086	0.034	0.575	0.0240	1.82
4-OH-PHE	0.001	89	0.030	0.021	0.004	0.090	0.00330	0.169	90	0.050	0.030	0.004	0.178	0.00605	0.396
1-OH-PY	0.002	86	0.106	0.076	0.009	0.308	0.0160	0.838	94	0.118	0.097	0.016	0.303	0.0366	0.461

Values are rounded to three significant figures and maximum three decimal places; LOQs limit of quantification (calculated with mean concentration of creatinine 1.40 mg/mL); for results below LOQ and with more than 50% positive samples, a value ½ of LOQ was used for calculation of arithmetic mean, median, 5th and 95th percentile. Abbreviations of analytes: naphthalene-1-ol (1-OH-NAP), naphthalene-2-ol (2-OH-NAP), fluorene-2-ol (2-OH-FL), phenanthrene-1-ol (1-OH-PHE), phenanthrene-2-ol (2-OH-PHE), phenanthrene-3-ol (3-OH-PHE), phenanthrene-4-ol (4-OH-PHE), phenanthrene-9-ol (9-OH-PHE), pyrene-1-ol (1-OH-PY), benzo[a]pyrene-3-ol (3-OH-BaP), chrysene-6-ol (6-OH-CHRY)

Out of 11 target analytes, only nine were found in quantifiable concentrations. The analytes 6-OH-CHR and 3-OH-BaP were not detected in any samples. The analyte 2-OH-NAP exhibited the highest concentrations of the mean (1st period, 3.07 µg/g creatinine; 2nd period, 3.99 µg/g creatinine) followed by its isomer 1-OH-NAP and fluorene-2-ol (2-OH-FL) (1st period, 0.309 and 0.530 µg/g creatinine; 2nd period, 0.312 and 0.332 µg/g creatinine, respectively).

**Fig. 1:**
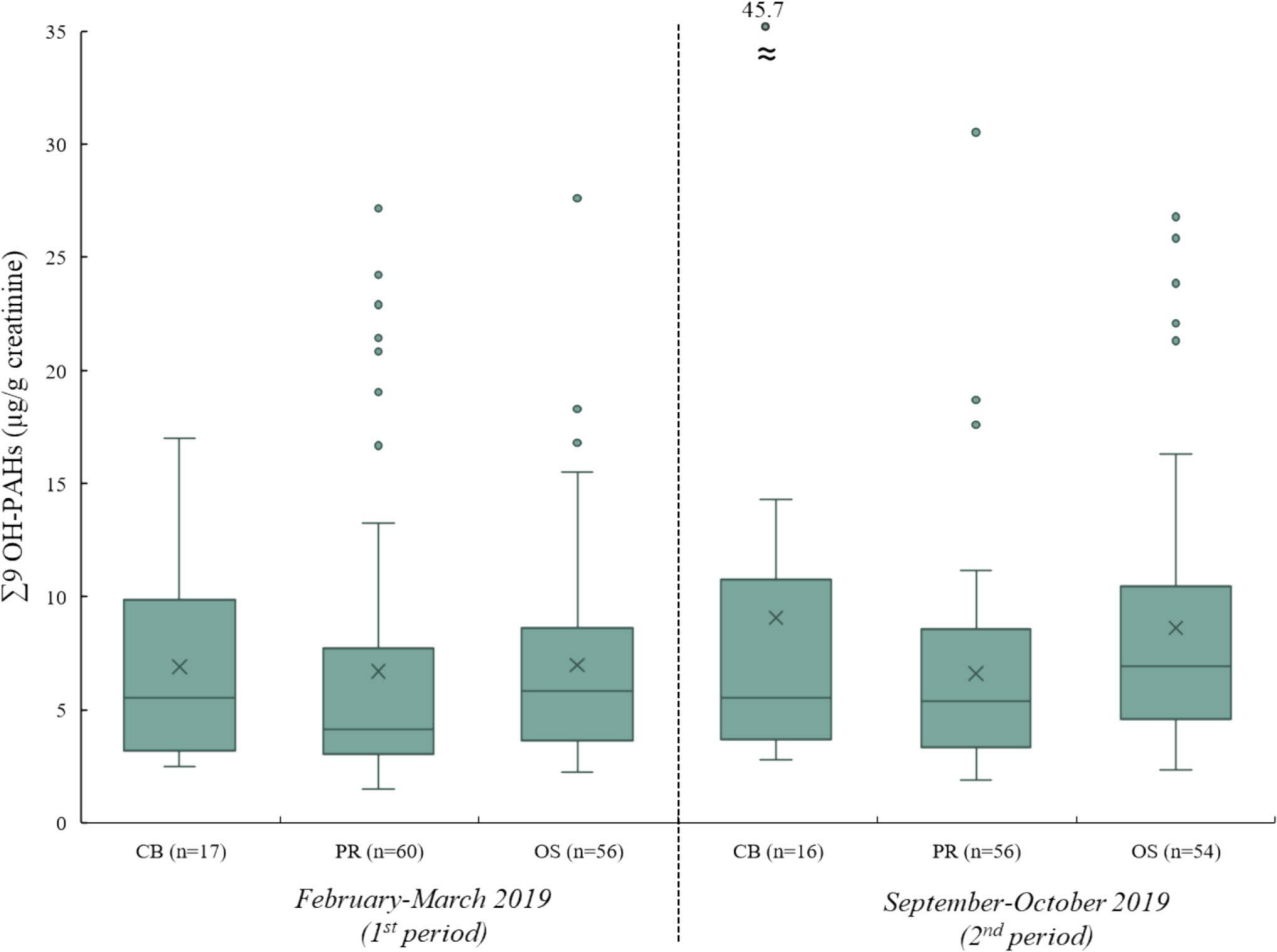
Comparison of urinary concentrations of ∑9 OH-PAHs sorted by demographic location in both sampling periods.* Note: CB = Ceske Budejovice, PR = Prague, OS = Ostrava; median = the horizontal line in the box; arithmetic mean = the cross in the box; 75th and 25th quartiles = the upper and lower limits of the box; 1.5 × the interquartile range = the length of the whiskers, outside dots = outliers; for results below LOQ and with more than 50% positive samples, a value ½ of LOQ was used*

Figure 1 shows the results of urinary concentration of the sum of the nine OH-PAHs (∑9 OH-PAHs) sorted by geographical location and sampling period.

Within the 1st sampling period, 133 urine samples from municipal police officers were analysed. The median concentration of ∑9 OH-PAHs (5th–95th percentile) in the urine samples from Ceske Budejovice (*n* = 17) was 5.52 µg/g creatinine (2.63–14.9 µg/g creatinine), from Prague (*n* = 60) 4.15 µg/g creatinine (1.99–21.5 µg/g creatinine) and from Ostrava (*n* = 56) 5.84 µg/g creatinine (2.41–15.8 µg/g creatinine). A total of 126 urine samples from the same municipal police officers were analysed in the 2nd sampling period. The median concentration of ∑9 OH-PAHs (5th–95th percentile) in the urine samples from Ceske Budejovice (*n* = 16) was 5.54 µg/g creatinine (2.90–22.2 µg/g creatinine), from Prague (*n* = 56) 5.37 µg/g creatinine (2.08–12.8 µg/g creatinine) and from Ostrava (*n* = 54) 6.92 µg/g creatinine (2.72–22.7 µg/g creatinine). No statistically significant difference in the measured concentrations of ∑9 OH-PAHs in urine was found between geographical locations in both sampling periods (one-way ANOVA test; detailed results are presented in Table S6).

### Concentrations of particulate phase PAHs in inhaled air by individuals

To assess the extent of exposure by inhalation, 20 PM2.5-bound PAHs were monitored in inhaled air by individual municipal police officers in three locations of the Czech Republic (1st period: *n* = 133; and 2nd period: *n* = 126) using personal samplers. The results of all participants sorted by sampling period are summarised in Table 4.

**Table 4:** Measured concentrations for each PM2.5-bound PAHs in inhaled air of individual (ng/m^3^) sorted by the sampling periods

ng/m^3^	LOQs	*February*–*March 2019* (1st period)—ALL PARTICIPANTS							*September*–*October 2019* (2nd period)—ALL PARTICIPANTS						
		*n* = 133							*n* = 126						
		Positive samples (%)	Arithmetic mean	Median	5th Percentile	95th Percentile	MIN	MAX	Positive samples (%)	Arithmetic mean	Median	5th Percentile	95th Percentile	MIN	MAX
PHE	0.050	51	0.075	0.052	0.025	0.228	0.051	0.725	68	0.173	0.113	0.025	0.505	0.052	0.622
AN	0.050	4	-	-	-	-	0.070	0.101	0	n.d	n.d	-	-		
FLA	0.050	75	0.114	0.085	0.025	0.278	0.050	0.670	63	0.132	0.080	0.025	0.376	0.050	1.06
PY	0.050	86	0.163	0.131	0.025	0.415	0.050	0.993	71	0.203	0.095	0.025	0.476	0.052	3.18
BcFL	0.050	3	-	-	-	-	0.096	0.296	0	n.d	n.d	-	-	-	-
BaA	0.050	83	0.143	0.106	0.025	0.288	0.050	1.77	67	0.117	0.089	0.025	0.316	0.055	0.517
CPP	0.050	96	0.345	0.244	0.054	0.778	0.052	5.09	77	0.157	0.122	0.025	0.392	0.057	1.10
CHR	0.050	88	0.179	0.129	0.025	0.373	0.058	1.55	81	0.180	0.128	0.025	0.483	0.053	0.675
5MC	0.050	0	n.d	n.d	-	-	-	-	0	n.d	n.d	-	-	-	-
BbF	0.050	96	0.343	0.256	0.077	0.748	0.069	4.38	97	0.357	0.274	0.061	0.979	0.052	1.38
BkF	0.050	83	0.170	0.120	0.025	0.383	0.053	2.72	75	0.171	0.130	0.025	0.476	0.050	0.699
BjF	0.050	86	0.188	0.130	0.025	0.421	0.051	2.53	80	0.248	0.185	0.025	0.627	0.053	0.954
BaP	0.050	97	0.321	0.231	0.078	0.752	0.057	5.18	96	0.378	0.274	0.053	0.976	0.050	1.67
IP	0.050	96	0.308	0.229	0.057	0.609	0.054	4.43	94	0.353	0.267	0.032	0.846	0.052	1.43
DBahA	0.050	18	-	-	-	-	0.055	1.27	53	0.082	0.060	0.025	0.213	0.051	0.369
BghiP	0.050	98	0.369	0.277	0.085	0.691	0.050	5.43	98	0.401	0.315	0.065	0.976	0.052	1.53
DBaIP	0.050	0	n.d	n.d	-	-	-	-	0	n.d	n.d	-	-	-	-
DBaeP	0.100	2	-	-	-	-	0.328	1.03	0	n.d	n.d	-	-	-	-
DBaiP	0.100	1	-	-	-	-	0.383	0.383	0	n.d	n.d	-	-	-	-
DBahP	0.100	0	n.d	n.d	-	-	-	-	0	n.d	n.d	-	-	-	-

Values are rounded to three significant figures and maximum three decimal places; LOQs limit of quantification; for results below LOQ and with more than 50% positive samples, a value ½ of LOQ was used for calculation of arithmetic mean, median, 5th and 95th percentile. Abbreviations of analytes: phenanthrene (PHE), anthracene (AN), fluoranthene (FLA), pyrene (PY), benzo[*a*]anthracene (BaA), chrysene (CHR), benzo[*b*]fluoranthene (BbF), benzo[*k*]fluoranthene (BkF), benzo[*a*]pyrene (BaP), dibenzo[*a,h*]anthracene (DBahA), indeno[1,2,3-*cd*]pyrene (IP), benzo[*g,h,i*]perylene (BghiP), benzo[*c*]fluorene (BcFL), cyclopenta[*c,d*]pyrene (CPP), 5-methylchrysene (5MCH), benzo[*j*]fluoranthene (BjF), dibenzo[*a,l*]pyrene (DBalP), dibenzo[*a,e*]pyrene (DBaeP), dibenzo[*a,i*]pyrene (DBaiP), dibenzo[*a,h*]pyrene (DBahP)

Out of 20 target analytes, only 17 were found in quantifiable concentrations. The analytes 5-methylchrysene (5MCH), dibenzo[*a,i*]pyrene (DBaiP) and DBahP were not detected in any samples. Only in ≤ 4% of all samples collected in the 1st period were the analytes anthracene (AN), BcFL, dibenzo[*a,e*]pyrene (DBaeP) and DBaiP found. According to the results from personal samplers, the municipal police officers, for whom analytes DBaeP and DBaiP were found in their filters, were exposed to the highest concentrations of Σ20 PAHs (19.0 and 36.0 ng/m^3^) from the entire group of participants. The individually measured concentrations of Σ20 PAHs ranged from 0.325 to 36.0 ng/m^3^.

Individual measured concentrations for the analyte benzo[*a*]pyrene (BaP) varied between < 0.050 and 5.19 ng/m^3^ for each sampling period and location. The European average emission limit value of 1 ng/m^3^ (*Standards-Air Quality-Environment-European Commission *n.d) was exceeded in four filter samples from personal air samplers of municipal police officers in Ostrava (1st period) and in five samples of filters from personal air samplers of municipal police officers in Prague (2nd period).

**Fig. 2:**
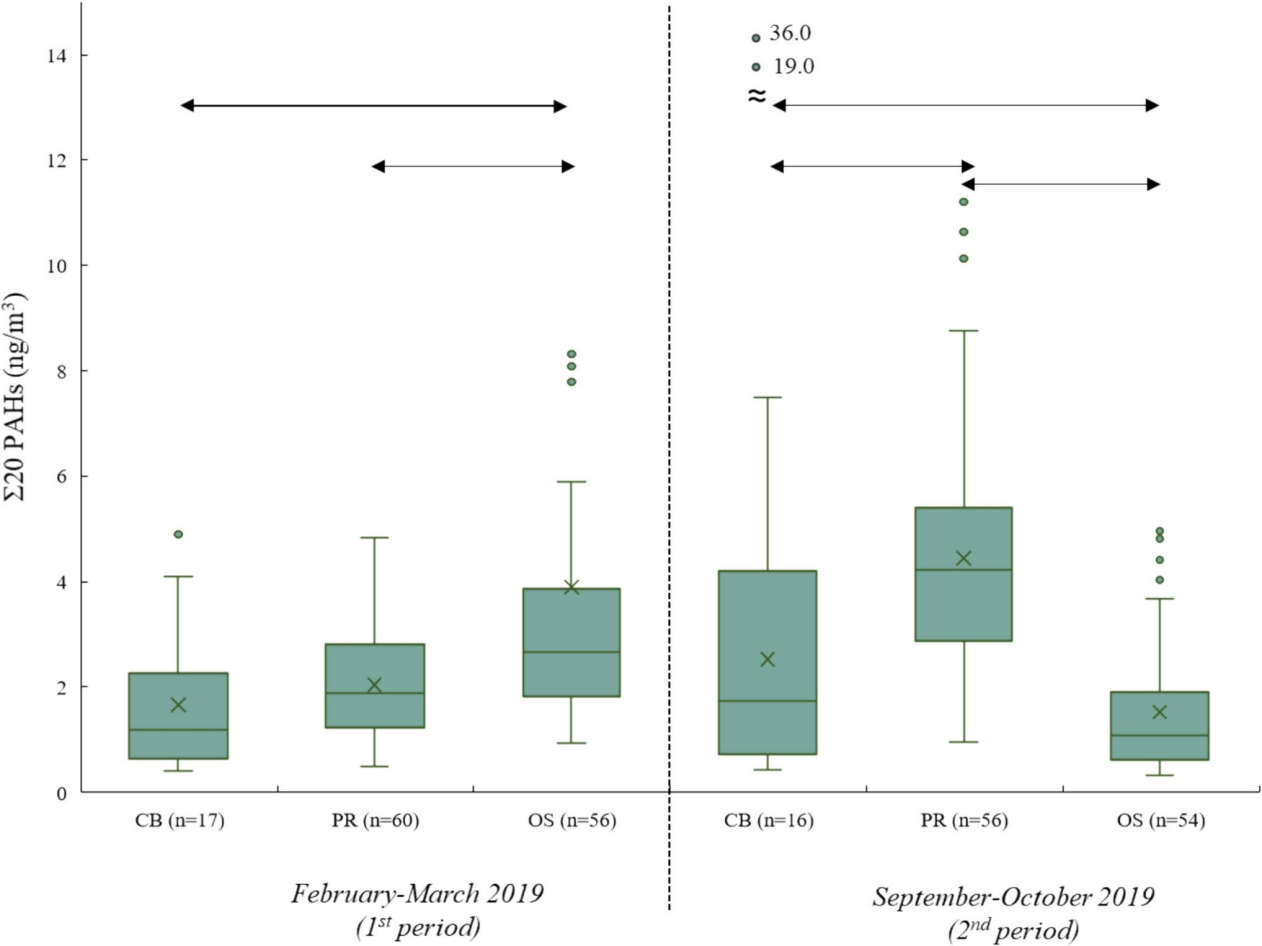
Comparison of concentrations of Σ20 PAHs in the inhaled air of municipal police officers sorted by demographic location and sampling period. *Note: CB = Ceske Budejovice, PR = Prague, OS = Ostrava; median = the horizontal line in the box; arithmetic mean = the cross in the box; 75th and 25th quartiles = the upper and lower limits of the box; 1.5 × the interquartile range = the length of the whiskers, outside dots = outliers; for results below LOQ and with more than 50% positive samples, a value ½ of LOQ was used;* *the arrows show statistically significant differences* *(q > q*_*crit*_*, α = 0.05)*

Figure 2 shows the results of Σ20 PAHs in the inhaled air of municipal police officers sorted by demographic location and sampling period.

In the 1st sampling period, the median ∑20 PAHs (5th–95th percentile) for samples from Ceske Budejovice (*n* = 17) was 1.19 ng/m^3^ (0.470–4.25 ng/m^3^), from Prague (*n* = 60) 1.89 ng/m^3^ (0.718–3.85 ng/m^3^) and from Ostrava (*n* = 56) 2.65 ng/m^3^ (1.43–8.15 ng/m^3^), while in the 2nd sampling period the median ∑20 PAHs (5th–95th percentile) for samples from Ceske Budejovice (*n* = 16) was 1.73 ng/m^3^ (0.496–6.52 ng/m^3^), from Prague was (*n* = 56) 4.23 ng/m^3^ (1.47–9.09 ng/m^3^) and from Ostrava (*n* = 54) was 1.09 ng/m^3^ (0.427–4.18 ng/m^3^).

## Discussion

Among the levels of OH-PAHs, no statistically significant differences between groups were found. The different metabolic efficiency of the substances in the organism can potentially explain the absence of significant differences between the regions. Another reason could be a process of epigenetic modification represented by a unique DNA methylation pattern previously described in a population from Ostrava chronically exposed to high levels of BaP (Honkova et al. 2022; Rossnerova et al. 2017, 2023).

Since the studied municipal police officers spent a large part of their shift outside, it was hypothesised that air pollution by industry and/or traffic might significantly influence the overall exposure of municipal police officers. For the first round of sampling, detailed information on the time schedule of participants was provided (see Table 2). The samples of urine and filters were divided into groups according to how long the police officers spent outside of the buildings and assessed whether there were statistically significant differences between the measured concentrations. Higher levels of ƩOH-PAHs/ƩPAHs were not observed in the urine/filters of police officers who spent a longer time outside during their shifts.

Based on the knowledge of air pollution in the selected locations (5-year average of annual BaP concentrations: Ceske Budejovice 0.4–2 ng/m^3^, Prague 0.6–2 ng/m^3^ and Ostrava > 2 ng/m^3^) (CHMI 2018), it was hypothesised that the municipal police officers will inhale the concentrations of PAHs during their shift in the order of Ostrava > Prague > Ceske Budejovice. For the 1st sampling period, the order of the cities according to the level of ∑20 PAHs concentrations inhaled by the municipal police officers was as follows: Ostrava* > Prague > Ceske Budejovice (*meets the Tukey-Kraer’s test criterion for a statistically significant difference (*q* > *q*_crit_, *α* = 0.05)). The detailed statistical analysis results are presented in Table S7 (one-way ANOVA) and Table S8 (Tukey–Kramer’s test). On the contrary, the weather conditions during the sampling day in Ostrava probably caused the lower measured concentrations of the target analytes in the samples from the 2nd period. They changed the order as follows: Prague* > Ceske Budejovice* > Ostrava* (*meets the Tukey–Kramer’s test criterion for a statistically significant difference (*q* > *q*_crit_, *α* = 0.05)).

Considering the respiratory risk associated with PM2.5-bound PAHs, ICR values indicate a high potential risk of cancer in 10.7% of samples in Ostrava (1st sampling round), in 12.5% of samples from CB and in 21.4% of samples from Prague (2nd sampling round).

The Pearson’s correlation coefficient was used to evaluate the relationship between the personal inhalation exposure to parent PAHs and their metabolites in the urine of individual police officers and short-term exposure for both (ƩOH-PAHs and ƩPAHs). Based on the spectrum of target analytes in this study, PHE and PYR with their metabolites Σ5 OH-PHE and 1-OH-PYR were selected. The calculated correlation coefficient *r*_s_, which ranged from − 0.124 to 0.194, showed a small, almost negligible relationship (Connelly 2012). No correlation was found between the duration of the time spent outdoor and the measured concentrations of ƩOH-PAHs and ƩPAHs.

In previously published studies looking at the relationship between urinary excretion of OH-PAHs and inhaled PAHs, correlations between PHE/PY and their metabolites were observed in workers with high occupational exposure and smokers (Grimmer et al. 1994; Merlo et al. 1998). However, different results have been demonstrated for non-smokers with non-occupational exposure. In a Portuguese study looking at the impact of indoor air on children in kindergartens exposure to PAHs, no apparent relationships (*r*_s_ ≤ 0.300) were found for 1-OH-PY and PY. Correlations were observed for low molecular weight naphthalene (NAP) and acenaphthene (AC) with 1-OH-NAP + 1-hydroxyacenaphthene (1-OH-AC) with *r*_s_ varying from 0.218 to 0.700 (Oliveira et al. 2017). In a Chinese study, university students’ dietary and inhalation exposure to PAHs was investigated. It was found that in addition to 1-OH-PY, metabolites of PHE can also be used as biomarkers for short-term intake of PAHs through diet. Still, for indicating exposure to high molecular weight (with three to seven aromatic rings), PAHs via inhalation 3-hydroxybenzo[*a*]anthracene (3-OH-BaA) should be used (Zhang et al. 2014).

Based on the above information, these results indicate that inhalation exposure is not a significant source of total exposure to PAHs for Czech municipal police officers. The other significant source of participant’s exposure to PAHs may be from their diet, but its assessment was not part of the study.

### Comparison of results from this study with those performed worldwide

The measured concentrations of OH-PAHs in the urine samples of municipal police officers were compared with other studies conducted worldwide (see Table 5). In general, traffic police officers are considered an occupationally exposed group of the population (Hu et al. 2007; Liu et al. 2007; Kamal et al. 2016). Only a few studies addressed urinary biomarker monitoring to assess traffic police officers’ exposure to PAHs (Merlo et al. 1998; Toriba and Hayakawa 2007; Kamal et al. 2016)*.* Given that the monitored municipal police officers were exposed to PAHs from the air and the roads for most of their working hours, they can be considered an occupationally exposed group of the population.

**Table 5:** Comparison of presented results of OH-PAHs in the urine with other studies

Country	Population group	Year of sampling	Number of samples	Unit	1-OH-NAP	2-OH-NAP	2-OH-FL	2-OH-PHE	3-OH-PHE	4-OH-PHE	1-OH-PHE	9-OH-PHE	1-OH-PY	Ref
Italy	**Traffic police officers**	1993–1994	89	Geometric mean (µmol/mol creatinine; µg/g creatinine*)	-	-	-	-	-	-	-	-	0.14 (0.27)	Merlo et al. 1998
	Control subjects (office workers)		43		-	-	-	-	-	-	-	-	0.09 (0.17)	
Thailand	Taxi drivers	n.s	10	Geometric mean (µg/g creatinine)	8.42	2.91	0.55	0.26	0.33	0.03	0.17		0.52	Toriba and Hayakawa 2007
	**Traffic police officers**	n.s	10		5.95	3.49	0.60	0.19	0.19	0.05	0.22		0.35	
USA	Airforce smokers (pre-shift)	2008	20	Geometric mean (µg/g creatinine)	3.45	4.63	0.44	0.06	0.10	0.02	n.a	0.12	0.09	Rodrigues et al. 2014
	Airforce smokers (post-shift)		20		6.01	6.38	0.682	0.06	0.11	0.02	n.a	0.12	0.07	
	Airforce non-smokers (pre-shift)		18		1.33	2.84	0.25	0.04	0.07	0.02	n.a	0.10	0.07	
	Airforce non-smokers (post-shift)		18		2.50	3.56	0.41	0.05	0.08	0.029	n.a	0.11	0.06	
USA	Firefighters (pre-shift)	2008–2009	14	Geometric mean (µg/g creatinine)	2.09	4.04	0.5	0.25	0.12	n.a	0.2	0.03	0.31	Adetona et al. 2017
	Firefighters (post-shift)		14		8.82	12.07	1.49	0.56	0.35	n.a	0.71	0.12	0.58	
Pakistan	**Traffic police officers**	2014	45	Geometric mean (µmol/mol creatinine µg/g creatinine*)	-	-	-	-	-	-	-	-	0.98 (1.89)	Kamal et al. 2016
	Driver workers		50		-	-	-	-	-	-	-	-	0.89 (1.72)	
	Control subjects		34		-	-	-	-	-	-	-	-	0.7 (1.35)	
Czech Republic	Women	2013–2014	265	Median (ng/mL urine; µg/g creatinine)	0.66 (0.54)	5.95 (5.36)	0.44 (0.37)	0.46 (0.42)	0.21 (0.20)	0.22 (0.18)	0.09 (0.09)	0.11 (0.11)	0.22 (0.21)	Urbancova et al. 2017
Czech Republic	Women	2016–2017	330	Median (ng/mL urine; µg/g creatinine)	0.36 (0.41)	4.62 (5.15)	0.23 (0.23)	0.24 (0.26)	0.15 (0.17)	0.40 (0.45)	0.06 (0.06)	0.15 (0.12)	0.11 (0.12)	Urbancova et al. 2020
Czech Republic	**Police officers**	2019	259	Median (ng/mL urine; µg/g creatinine)	0.61 (0.41)	4.87 (3.41)	0.44 (0.34)	0.12 (0.09)	0.14 (0.10)	0.03 (0.03)	0.27 (0.20)	0.10 (0.07)	0.12 (0.08)	Presented study

*n.s.* not specified

^*^µg/g creatinine calculated from µmol/mol creatinine with molar mass for 1-OH-PY 218.25 g/mol and creatinine 113.2 g/mol. Abbreviations of analytes: naphthalene-1-ol (1-OH-NAP), naphthalene-2-ol (2-OH-NAP), fluorene-2-ol (2-OH-FL), phenanthrene-1-ol (1-OH-PHE), phenanthrene-2-ol (2-OH-PHE), phenanthrene-3-ol (3-OH-PHE), phenanthrene-4-ol (4-OH-PHE), phenanthrene-9-ol (9-OH-PHE), pyrene-1-ol (1-OH-PY), benzo[a]pyrene-3-ol (3-OH-BaP), chrysene-6-ol (6-OH-CHRY)

Thus, the worldwide studies selected for the comparison are focused on occupationally exposed individuals. To better understand the exposure of the Czech population, two studies that focus on the analysis of OH-PAHs in urine samples collected from Czech women were also included in the comparison (see below).

As shown in Table 5, the urinary metabolites of NAP, namely 1- and 2-OH-NAP, were the most dominant compounds in all the studies compared. The concentrations of the other target compounds were variable. The PAH concentrations Czech police officers presented in this study are exposed to are at the same level as the general population of the Czech Republic, represented here by Czech women studied in 2013–2014 (Urbancova et al. 2017) and 2016–2017 (Urbancova et al. 2020).

The overall concentration of all monitored OH-PAHs measured in urine samples from Czech police officers presented in this study is comparable to the results published by Rodrigues et al. (2014), specifically with the concentrations of OH-PAHs measured in urine samples from US Airforce workers who smoke, with samples collected pre-shift and with the concentrations of OH-PAHs in urine samples from US Airforce workers who do not smoke, collected post-shift. Similar concentrations of the target compounds were also measured in urine samples from US firefighters (pre-shift) (Adetona et al. 2017).

It can be concluded that Czech police officers are not exposed to such high concentrations of PAHs compared to other highly exposed occupations, e.g. asphalt workers (Buratti et al. 2007), steel workers (Onyemauwa et al. 2009) and coke-oven workers (Campo et al. 2010).

To compare personal inhalation PAH exposure between PAH concentrations determined in this study and previously published data, only studies in which personal air samplers were used were considered and are summarised in Table 6. Since the total number of analytes monitored and the results presented (concentrations in gaseous and/or particulate phase) differ across the studies, the analyte BaP was selected to compare personal inhalation exposure of different population groups.

**Table 6:** Comparison of presented results of PAHs in the filters from personal air samplers with other studies

Country	Population group	Number of samples	Unit	Sampling period	BaP (ng/m^3^)	ΣPAHs (ng/m^3^)		Ref
Finland	Bus garage workers	5 air samples from each worker (*n* = 22) per season	Arithmetic mean	Winter	2.9 (ND–60)	25 (ND–494)^c^	FL, PHE, AN, FLA, PY, BaA, CHR, BbF, BkF, BaP, DBahA, BghiP and IP	Kuusimaki et al. 2003
				Summer	0.6 (ND–8.9)	8.5 (ND–46)^c^		
Italy	Asphalt workers (AW) and roadside construction workers (CW)	AW = 100	Median (min–max)	Spring–Summer	AW = 0.33 (< 0.03–40.25)	AW = 607 (127–2973)^a^	*NAP, AC,* FL, PHE, AN, FLA, PY, BaA, CHR, BbF, BkF, BaP, DBahA, BghiP and IP	Campo et al. 2006
		CW = 47			CW = 0.61 (0.04–2.71)	CW = 405 (157–940)^a^		
China—Haidian District	Traffic police officers	3 air samples per day (10 days in total)	Mean	Winter	51.9	4300^b^ and 750^c^	*NAP, ACL, AC,* FL, PHE, AN, FLA, PY, BaA, CHR, BbF, BkF, BaP, DBahA, BghiP and IP	Liu et al. 2007
China—Taianjin	Traffic policemen	10	Mean	Summer	26.2	867.5^a^	FL, PHE, AN, FLA, PY, BaA, CHR, BbF, BkF, BaP, DBahA, BghiP and IP	Hu et al. 2007
China	Rural home with biomass fuelled cooking: cooking person (CP) + control group in the house (CG)	24	Mean	Winter	CP = 190 and CG = 46	CP = 1610 and CG = 684^c^	*ACL, AC,* FL, PHE, AN, FLA, PY, BaA, CHR, BbF, BkF, BaP, DBahA, BghiP and IP	Ding et al. 2012
				Summer	CP = 1.2 and CG = 1.3	n.s		
China—Taiyuan	Volunteers from rural (RA) and urban areas (UA)	126	Median (interquartile range)	Heating season	RA = 30.8 (12.3–48.3) and UA = 18.9 (11.5–35)	RA = 770 (504–1071) and UA = 695 (540–1051)^a^	*ACL, AC,* FL, PHE, AN, FLA, PY, BaA, CHR, BbF, BkF, BaP, DBahA, BghiP and IP	Duan et al. 2014
				Non-heating season	RA = 0.101 (0.0913–1.86) and UA = 8.26 (4.74–13.7)	RA = 312 (201–412) and UA = 404 (266–544)^a^		
Czech Republic	Police officers from three different localities: Ceske Budejovice (CB), Prague (PR) and Ostrava (OS)	259	Median (interquartile range)	February–March	CB = 0.165 (0.0250–0.634), PR = 0.200 (0.0813–0.472) and OS = 0.287 (0.121–1.13)	CB = 1.19 (0.470–4.25), PR = 1.89 (0.718–3.85) and OS = 2.65 (1.43–8.15)^c^	PHE, AN, FLA, PY, BcFL BaA*, CPP,* CHR, 5MC, BbF, BkF, *BjF*, BaP, IP DBahA, BghiP, DBaIP, DBaeP, DBaiP, DBahP	Presented study
				September–October	CB = 0.199 (0.0687–0.855), PR = 0.563 (0.178–1.25) and OS = 0.110 (0.0250–0.508)	CB = 1.73 (0.496–6.52), PR = 4.23 (1.47–9.09) and OS = 1.09 (0.427–4.18)^c^		

*ND* not detected

^a^Gaseous and particulate phase

^b^Gaseous phase

^c^Particulate phase

Abbreviations of analytes: fluorene (FL), naphthalene (NAP), acenaphthene (AC), acenaphthylene (ACL), phenanthrene (PHE), anthracene (AN), fluoranthene (FLA), pyrene (PY), benzo[*a*]anthracene (BaA), chrysene (CHR), benzo[*b*]fluoranthene (BbF), benzo[*k*]fluoranthene (BkF), benzo[*a*]pyrene (BaP), dibenzo[*a,h*]anthracene (DBahA), indeno[1,2,3-*cd*]pyrene (IP), benzo[*g,h,i*]perylene (BghiP), benzo[*c*]fluorene (BcFL), cyclopenta[*c,d*]pyrene (CPP), 5-methylchrysene (5MCH), benzo[*j*]fluoranthene (BjF), dibenzo[*a,l*]pyrene (DBalP), dibenzo[*a,e*]pyrene (DBaeP), dibenzo[*a,i*]pyrene (DBaiP), dibenzo[*a,h*]pyrene (DBahP)

Two of these studies focus on the occupational group of traffic police officers from a typical metropolis in China (Hu et al. 2007; Liu et al. 2007). The mean BaP concentrations in these studies were 51.9 and 26.2 ng/m^3^, respectively, and were approximately 100 × greater than the data in this study. Another study that looked at personal inhalation exposure to PAHs in urban and rural residents during the heating (January–February) and non-heating (September–October) seasons was conducted in China by Duan et al. (2014). Compared to the presented study, BaP concentrations in personal air samples are similar to volunteers from rural areas during the non-heating season (median, 0.101 ng/m^3^) but much lower than another group of volunteers from urban areas during both heating and non-heating seasons (median 18.9 and 8.26 ng/m^3^).

An Italian study by Campo et al. (2006) addresses the assessment of inhalation exposure of asphalt and road construction workers to PAHs. The median concentration of BaP for asphalt workers was 0.33 ng/m^3^, similar to the presented data. Another European study published by Kuusimaki et al. (2003) focused on the analysis of PAHs originating from diesel engine emissions in the personal air samples of bus garage workers. The measured BaP concentrations in samples from summer (mean 0.6 ng/m^3^) were comparable to the data from this study collected from police officers from Prague in September–October.

## Conclusion

This study provides information on the impact of air pollution on the exposure of Czech municipal police officers to PAHs during two seasons (February–March 2019 (1st period) and September–October 2019 (2nd period)). In the 1st period, statistically significant (*α* = 0.05) higher concentrations of ∑20 PM2.5-bound PAHs were measured in inhaled air of municipal police officers from Ostrava (air polluted location) compared to those from Prague (capital city) and Ceske Budejovice (control location). These results correlate with these locations’ average annual air pollution (CHMI 2018). The 2nd period was affected by windy conditions in Ostrava on the sampling days; therefore, the PAH concentrations in the sampled air were lower than those in other sampling areas. No statistically significant differences in the concentrations of OH-PAHs in the urine of police officers were found.

The results of this study indicate that inhalation exposure is not a major source of the total body burden of PAHs for Czech municipal police officers. The significant source of exposure may be diet. Czech police officers presented are exposed to the same levels of PAHs as the general population of the Czech Republic.

## Supplementary Information

Below is the link to the electronic supplementary material.

Supplementary file1 (DOCX 79.6 KB)

## Data Availability

All data are available in our paper or from the corresponding author on reasonable request.
