# Supplementary material for: Biomarkers of exposure to polycyclic aromatic hydrocarbons in urine of municipal police officers: impact of inhalation on total exposure
Source: Environ Sci Pollut Res Int. 2025 Apr 8;32(17):11097–113. doi: 10.1007/s11356-025-36342-2 (PMC12014768; doi:10.1007/s11356-025-36342-2)
Supplement: Supplementary file 1 — Supplementary file1 (DOCX 79.6 KB) [file 11356_2025_36342_MOESM1_ESM.docx]

**JOURNAL OF ENVIRONMENTAL SCIENCE AND POLLUTION RESEARCH**

**Electronic Supplementary Material**

BIOMARKERS OF EXPOSURE TO POLYCYCLIC AROMATIC HYDROCARBONS IN URINE OF MUNICIPAL POLICE OFFICERS: IMPACT OF INHALATION ON TOTAL EXPOSURE

Veronika Gomersall^1^, Katerina Ciglova^1^, Ondrej Parizek^1^, Andrea Rossnerova^2^, Pavel Rossner Jr.^2^, Radim J. Sram†^3^, Jan Topinka^2^, Jana Pulkrabova^1^*

^1^ University of Chemistry and Technology, Prague, Faculty of Food and Biochemical Technology, Department of Food Analysis and Nutrition, Technicka 3, 166 28 Prague 6, Czech Republic

^2^ Institute of Experimental Medicine AS CR, Department of Toxicology and Molecular Epidemiology, Videnska 1083, 142 20 Prague 4, Czech Republic

^3^ Institute of Experimental Medicine AS CR, Department of Genetic Toxicology and Epigenetics, Videnska 1083, 142 20 Prague 4, Czech Republic

E-mail: [jana.pulkrabova@vscht.cz](mailto:jana.pulkrabova@vscht.cz)

† deceased 2022-10-29

**UHPLC-MS/MS analysis of 11 OH-PAHs**

UHPLC analysis of 11 OH-PAHs in urine was performed using an Agilent 1290 Infinity II LC system. The analytes were separated on a PFP (pentafluorophenyl) Kinetex column, Phenomenex, (USA) (100 mm × 2.1 mm × 1.7 μm). The used mobile phases were (A) water and (B) methanol; more details are mentioned in an article by Lankova et al., 2016. The total run time for each injection was 13 minutes. The UHPLC system was coupled to a QTRAP® 6500+ mass spectrometer (Sciex, Canada) with electrospray ionisation in negative ion mode (ESI-, capillary voltage -4.5 kV, desolvation temperature 500 °C). The instrument was operated in Multiple Reaction Monitoring (MRM) mode (quantitative and qualitative transitions are shown in **Table S3**).

**GC-MS/MS analysis of 20 PAHs**

The GC-MS/MS analysis of 20 PAHs was performed using a 7890A gas chromatograph with a 7000C triple quadrupole MS system (both Agilent, USA) in electron ionisation (EI) mode. The analytes were separated using a capillary column Rxi^®^-PAH Restek, (USA) (40 m × 0.18 mm i.d. × 0.07 µm). A more detailed description of the GC-MS/MS method is stated in the following studies published by *Kalachova et al., 2013* and *Pulkrabova et al., 2016.*

*References*

Kalachova K, Pulkrabova J, Cajka T, Drabova L, Stupak M, Hajslova J (2013) Gas chromatography–triple quadrupole tandem mass spectrometry: a powerful tool for the (ultra)trace analysis of multiclass environmental contaminants in fish and fish feed. Anal. Bioanal. Chem. 405:7803-7815.

Pulkrabova J, Stupak M, Svarcova A, Rossner P, Rossnerova A, Ambroz A, Sram R, Hajslova J (2016) Relationship between atmospheric pollution in the residential area and concentrations of polycyclic aromatic hydrocarbons (PAHs) in human breast milk. Sci. Total Environ. 562:640-647.

**Table S1:** Information about used certified standards

| **Name** | **Abbreviation** | **CAS number** | **Manufactures** |
| --- | --- | --- | --- |
| creatinine (anhydrous) | - | 60-27-5 | Sigma-Aldrich (USA) |
| naphthalene-1-ol | 1-OH-NAP | 90-15-3 | Absolute Standards, Inc. (USA) |
| naphthalene-2-ol | 2-OH-NAP | 135-19-3 |  |
| fluorene-2-ol | 2-OH-FL | 2443-58-5 | Toronto Research Chemicals, Inc. (Canada) |
| phenanthrene-1-ol | 1-OH-PHE | 2433-56-9 |  |
| phenanthrene-2-ol | 2-OH-PHE | 605-55-0 |  |
| phenanthrene-3-ol | 3-OH-PHE | 605-87-8 |  |
| phenanthrene-4-ol | 4-OH-PHE | 7651-86-7 | Neochema (Germany) |
| phenanthrene-9-ol | 9-OH-PHE | 484-17-3 |  |
| pyrene-1-ol | 1-OH-PY | 5315-79-7 |  |
| benzo[*a*]pyrene-3-ol | 3-OH-BaP | 13345-21-6 |  |
| chrysene-6-ol | 6-OH-CHRY | 37515-51-8 | AccuStandard^®^ (USA) |
| [^2^H]_7_-naphtalene-1-ol | d_7_-1-OH-NAP | 124251-84-9 | Toronto Research Chemicals, Inc. (Canada) |
| [^2^H]_7_-naphtalene-2-ol | d_7_-2-OH-NAP | 78832-54-9 |  |
| [^2^H]_9_-fluorene-2-ol | d_9_-2-OH-FL | 922510-18-7 |  |
| [^2^H]_9_-phenanthrene-1-ol | d_9_-1-OH-PHE | 922510-23-4 |  |
| [^2^H]_9_-phenanthrene-2-ol | d_9_-2-OH-PHE | 922510-19-8 |  |
| [^2^H]_9_-phenanthrene-3-ol | d_9_-3-OH-PHE | 922510-20-1 |  |
| [^2^H]_8_-phenanthrene-9-ol | d_8_-9-OH-PHE | n.s. |  |
| [^2^H]_9_-pyrene-1-ol | d_9_-1-OH-PY | 132603-37-3 |  |
| [^2^H]_11_-benzo[*a*]pyrene-3-ol | d_11_-3-OH-BaP | 1246819-35-1 |  |
| phenanthrene* | PHE | 85-01-8 |  |
| anthracene* | AN | 120-12-7 |  |
| fluoranthene* | FLA | 206-44-0 |  |
| pyrene* | PY | 129-00-0 |  |
| benzo[*a*]anthracene* | BaA | 56-55-3 |  |
| chrysene* | CHR | 218-01-9 |  |
| benzo[*b*]fluoranthene* | BbF | 205-99-2 |  |
| benzo[*k*]fluoranthene* | BkF | 207-08-9 |  |
| benzo[*a*]pyrene* | BaP | 50-32-8 |  |
| dibenzo[*a,h*]anthracene* | DBahA | 53-70-3 |  |
| indeno[1,2,3-*cd*]pyrene* | IP | 193-39-3 | Dr. Ehrenstorfer, GmbH (Germany) |
| benzo[*g,h,i*]perylene* | BghiP | 191-24-2 |  |
| benzo[*c*]fluorene | BcFL | 205-12-9 |  |
| cyclopenta[*c,d*]pyrene | CPP | 27208-37-3 |  |
| 5-methylchrysene | 5MCH | 3697-24-3 |  |
| benzo[*j*]fluoranthene | BjFA | 205-82-3 |  |
| dibenzo[*a,l*]pyrene | DBalP | 191-30-0 |  |
| dibenzo[a,e]pyrene | DBaeP | 192-65-4 |  |
| dibenzo[*a,i*]pyrene | DBaiP | 189-55-9 |  |
| dibenzo[*a,h*]pyrene | DBahP | 189-64-0 |  |
| ^13^C_6_-phenanthrene | ^13^C_6_-PHE | 1189955-53-0 | Cambridge Isotope Laboratories, Inc. (USA) |
| ^13^C_6_-anthracene | ^13^C_6_-AN | 189811-60-7 |  |
| ^13^C_6_-fluoranthene | ^13^C_6_-FLA | 917378-10-0 |  |
| ^13^C_3_-pyrene | ^13^C_3_-PY | 1346601-04-4 |  |
| ^13^C_6_-benzo[*a*]anthracene | ^13^C_6_-BaA | 917378-11-1 |  |
| ^13^C_6_-chrysene | ^13^C_6_-CHR | n.s. |  |
| ^13^C_6_-benzo[*b*]fluoranthene | ^13^C_6_-BbF | 1397206-82-4 |  |
| ^13^C_6_-benzo[*k*]fluoranthene | ^13^C_6_-BkF | 1397194-60-3 |  |
| ^13^C_4_-benzo[*a*]pyrene | ^13^C_4_-BaP | 1346601-04-4 |  |
| ^13^C_6_-dibenzo[*a,h*]anthracene | ^13^C_6_-DBahA | 13250-98-1 |  |
| ^13^C_6_-indeno[1,2,3-*cd*]pyrene | ^13^C_6_-IP | [362044-56-2](https://www.lgcstandards.com/IT/en/search?text=362044-56-2) |  |
| ^13^C_6_-benzo[*g,h,i*]perylene | ^13^C_6_-BghiP | n.s. |  |
| ^13^C_6_-benzo[*c*]fluorene | ^13^C_6_-BcFL | 205-12-9 |  |
| ^13^C_6_-cyclopenta[*c,d*]pyrene | ^13^C_6_-CPP | n.s. |  |
| ^13^C_6_-5-methylchrysene | ^13^C_6_-5MCH | n.s. |  |
| ^13^C_6_-benzo[*j*]fluoranthene | ^13^C_6_-BjF | 1397194-60-3 |  |
| ^13^C_6_-dibenzo[*a,l*]pyrene | ^13^C_6_-DBalP | n.s. |  |
| ^13^C_6_-dibenzo[*a,e*]pyrene | ^13^C_6_-DBaeP | n.s. |  |
| ^13^C_12_-dibenzo[*a,i*]pyrene | ^13^C_12_-DBaiP | n.s. |  |
| ^13^C_6_-dibenzo[*a,h*]pyrene | ^13^C_6_-DBahP | n.s. |  |

*Note: n.s. …not specified, * PAH MIX 9.*

**Table S2:** Information about chemicals and other used materials

| **Category** |  | **Additional information** | **Manufactures** |
| --- | --- | --- | --- |
| Solvents | Ethyl acetate | Chromasolv ™ | Honeywell (USA) |
|  | Dichloromethane | HPLC gradient quality | Merck (Germany) |
|  | *n*-hexane |  |  |
|  | Isooctane |  |  |
|  | Methanol |  |  |
|  | Ultrapure water | Milli-Q® Integral System, TOC ≤ 5 μg∙L⁻¹ | Merck (Germany) |
| Chemicals | Picric acid | Moistened with water ≥98% | Sigma Aldrich (Germany) |
| Enzyme | β-glucuronidase | Type HP-2, glucuronidase activity ≥ 100 000 units/mL, sulfatase activity ≤ 7 500 units/mL |  |
| Sorbent | Supel^TM^ QuE Z-Sep |  |  |
| Other material | Microtubes with Mini-spin filters | Fisherbrand 1.5 mL; polyvinylidene fluoride, 0.2 µm | Ciro Manufacturing Corporation (USA) |
|  | Filters | Non-sterile, polytetrafluoroethylene, 5.0 µm, Ø 25 mm | Rotilabo ^®^ (Germany) |
|  | 96-well microtiter plates |  | Gama Group (Czech Republic) |
| Gages | Carbon dioxide | purity 3.0 | Siad a.s. (Czech Republic) |
|  | Helium | purity 6.0 |  |
|  | Nitrogen | purity 4.0 and 5.0 |  |

**Table S3:** Retention times and MS/MS parameters for determination of OH-PAHs

| **Analyte** | **Retention Time (min)** | **Quantification MRM transition** | **Declustering Potential (V)** | **Entering Potential (V)** | **Collision Energy (V)** | **Cell Exit Potential (V)** |
| --- | --- | --- | --- | --- | --- | --- |
|  |  | **Confirmation MRM transition** |  |  |  |  |
| **2-OH-NAP** | 4.3 | **143>115** | -10 | -10 | -34 | -13 |
|  |  | 144>116 | -10 | -10 | -34 | -13 |
| **1-OH-NAP** | 4.6 | **143>115** | -10 | -10 | -34 | -13 |
|  |  | 144>116 | -10 | -10 | -34 | -13 |
| **2-OH-FL** | 5.7 | **181>180** | -55 | -10 | -28 | -15 |
|  |  | 181>153 | -55 | -10 | -28 | -15 |
| **2-OH-PHE** | 6.2 | **193>165** | -50 | -10 | -40 | -9 |
|  |  | 194>164 | -50 | -10 | -40 | -9 |
| **3-OH-PHE** | 6.3 | **193>165** | -50 | -10 | -40 | -9 |
|  |  | 194>166 | -50 | -10 | -40 | -9 |
| **1-OH-PHE** | 6.4 | **193>165** | -50 | -10 | -40 | -9 |
|  |  | 194>166 | -50 | -10 | -40 | -9 |
| **9-OH-PHE** | 6.5 | **193>165** | -50 | -10 | -40 | -9 |
|  |  | 194>166 | -50 | -10 | -40 | -9 |
| **4-OH-PHE** | 6.7 | **193>165** | -130 | -10 | -38 | -19 |
|  |  | 193>41 | -130 | -10 | -70 | -19 |
| **1-OH-PY** | 7.5 | **217>189** | -40 | -10 | -46 | -17 |
|  |  | 218>190 | -40 | -10 | -50 | -19 |
| **6-OH-CHR** | 7.9 | **243>215** | -155 | -10 | -44 | -13 |
|  |  | 244>216 | -155 | -10 | -44 | -13 |
| **3-OH-BaP** | 8.6 | **267>239** | -120 | -10 | -48 | -21 |
|  |  | 268>240 | -120 | -10 | -48 | -21 |
| **d_7_-2-OH-NAP** | 4.3 | **150>122** | -85 | -10 | -36 | -15 |
|  |  | 151>123 | -85 | -10 | -36 | -15 |
| **d_7_-1-OH-NAP** | 4.5 | **150>122** | -85 | -10 | -36 | -15 |
|  |  | 151>122 | -85 | -10 | -54 | -15 |
| **d_9_-2-OH-FL** | 5.6 | **190>162** | -35 | -10 | -36 | -17 |
|  |  | 190>188 | -35 | -10 | -36 | -17 |
| **d_9_-2-OH-PHE** | 6.1 | **202>174** | -130 | -10 | -42 | -19 |
|  |  | 203>175 | -130 | -10 | -42 | -19 |
| **d_9_-3-OH-PHE** | 6.2 | **202>174** | -115 | -10 | -42 | -19 |
|  |  | 202>172 | -130 | -10 | -46 | -15 |
| **d_9_-1-OH-PHE** | 6.3 | **202>174** | -130 | -10 | -42 | -19 |
|  |  | 202>175 | -130 | -10 | -42 | -19 |
| **d_8_-9-OH-PHE** | 6.4 | **201>173** | -125 | -10 | -46 | -19 |
|  |  | 202>174 | -125 | -10 | -46 | -19 |
| **d_9_-1-OH-PY** | 7.5 | **226>198** | -135 | -10 | -52 | -19 |
|  |  | 227>199 | -135 | -10 | -52 | -19 |
| **d_11_-3-OH-BaP** | 8.5 | **278>250** | -150 | -10 | -50 | -27 |
|  |  | 279>251 | -150 | -10 | -50 | -27 |

*Note:* ***bold****… transitions for quantification; MRM…multiple reaction monitoring.*

**Table S4:** Performance characteristics of the UHPLC-MS/MS method

| **Analyte** | **LOQ** | **Certified value** | **Measured value** | **Recovery** | **RSD** |
| --- | --- | --- | --- | --- | --- |
|  | **(ng/mL urine)** | **(ng/mL urine)** | **(ng/mL urine)** | **(%)** | **(%)** |
| *SRM 3673 - Organic Contaminants in Non-Smokers´ Urine (n=6)* | | | | | |
| **2-OH-NAP** | 0.001 | 1.35 ± 0.03 | 1.43 ± 0.09 | 106 | 6 |
| **1-OH-NAP** | 0.002 | 211 ± 34 | 146 ± 12 | 70 | 8 |
| **2-OH-FL** | 0.001 | 0.107 ± 0.007 | 0.106 ± 0.016 | 99 | 15 |
| **2-OH-PHE** | 0.001 | 0.0247 ± 0.0043 | 0.0267 ± 0.0041 | 108 | 15 |
| **3-OH-PHE** | 0.001 | 0.0276 ± 0.0014 | 0.0278 ± 0.003 | 101 | 11 |
| **1-OH-PHE** | 0.002 | 0.0488 ± 0.0075 | 0.0514 ± 0.0063 | 105 | 12 |
| **9-OH-PHE** | 0.005 | 0.0116 ± 0.0009 | 0.0134 ± 0.0015 | 115 | 11 |
| **4-OH-PHE** | 0.001 | 0.0104 ± 0.001 | 0.0088 ± 0.0005 | 84 | 5 |
| **1-OH-PY** | 0.002 | 0.0305 ± 0.0018 | 0.0307 ± 0.0023 | 101 | 8 |
| **Creatinine (mg/kg)** | 132 | 505 ± 2 | 477 ± 9 | 95 | 2 |
| *artificially contaminated urine blank sample (n=6)* | | | | | |
| **6-OH-CHR^a^** | 0.005 | n. d. | 0.0475 ± 0.0062 | 95 | 13 |
| **3-OH-BaP^b^** | 0.05 | n. d. | 0.485 ± 0.078 | 97 | 16 |

*Note: LOQ…limit of quantification, RSD…a repeatability expressed as a relative standard deviation, ^a^…validation level 0.05 ng/mL urine, ^b^…validation level 0.5 ng/mL urine.*

**Table S5:** Performance characteristics of the GC-MS/MS method

| **Analyte** | **LOQ** | **Recovery** | **RSD** |
| --- | --- | --- | --- |
|  | **(ng/filter)** | **(%)** | **(%)** |
| *artificially contaminated unused air sampling filters (n=6) on validation level 10 ng/filter* | | | |
| PHE | 0.1 | 68 | 19 |
| AN | 0.1 | 67 | 18 |
| FLT | 0.1 | 78 | 17 |
| PY | 0.1 | 78 | 17 |
| BcFl | 0.1 | 87 | 12 |
| BaA | 0.1 | 78 | 15 |
| CPP | 0.1 | 96 | 21 |
| CHR | 0.1 | 79 | 17 |
| 5MCH | 0.1 | 82 | 21 |
| BbFA | 0.1 | 81 | 15 |
| BkFA | 0.1 | 75 | 19 |
| BjFA | 0.1 | 77 | 18 |
| BaP | 0.1 | 81 | 15 |
| IP | 0.1 | 81 | 16 |
| DBahA | 0.1 | 77 | 14 |
| BghiP | 0.1 | 82 | 16 |
| DBaIP | 0.3 | 94 | 18 |
| DBaeP | 0.3 | 95 | 21 |
| DBaiP | 0.3 | 93 | 18 |
| DBahP | 0.3 | 91 | 16 |

*Note: LOQ…limit of quantification, RSD…a repeatability expressed as a relative standard deviation.*

**Table S6**: Results from a one-way ANOVA test (α=0.05) for log10 concentration of OH-PAHs.

| **Compared parameter** | ***1^st^ period:* differences between location** | ***2^nd^ period:* differences between location** | ***Ceske Budejovice:* differences between sampling periods** | ***Prague:* differences between sampling periods** | ***Ostrava:* differences between sampling periods** |
| --- | --- | --- | --- | --- | --- |
| **p-value** | 0.509 | 0.105 | 0.561 | 0.527 | 0.0934 |
| **F value** | 0.678 | 2.30 | 0.345 | 0.404 | 2.86 |
| **F-crit** | 3.07 | 3.07 | 4.16 | 3.92 | 3.93 |
| **Statistically significant difference** | NO | NO | NO | NO | NO |

*Note: The statistically significant difference between compared geographical locations or sampling periods was if F > F _crit_ and p < α (α=0.05).*

*1^st^ sampling period = February – March 2019; 2^nd^ sampling period = September – October 2019.*

**Table S7**: Results from a one-way ANOVA test (α=0.05) for log10 concentration of PAHs

| **Compared parameter** | ***1^st^ period:* differences between location** | | ***2^nd^ period:* differences between location** | | ***Ceske Budejovice*: differences between sampling periods** | | ***Prague:* differences between sampling periods** | | ***Ostrava:* differences between sampling periods** | |  |
| --- | --- | --- | --- | --- | --- | --- | --- | --- | --- | --- | --- |
|  | **Σ20 PAHs** | **BaP** | **Σ20 PAHs** | **BaP** | **Σ20 PAHs** | **BaP** | **Σ20 PAHs** | **BaP** | **Σ20 PAHs** | **BaP** |  |
| **p-value** | 8.1E-07 | 2.4E-04 | 1.8E-15 | 1.0E-16 | 3.2E-01 | 2.2E-01 | 1.8E-12 | 8.9E-15 | 1.6E-10 | 3.6E-08 |  |
|  |  |  |  |  |  |  |  |  |  |  |  |
| **F value** | 15.7 | 8.89 | 45.6 | 50.4 | 1.0 | 1.5 | 62.6 | 79.6 | 50.2 | 35.2 |  |
|  |  |  |  |  |  |  |  |  |  |  |  |
| **F-crit** | 3.07 | 3.07 | 3.07 | 3.07 | 4.16 | 4.16 | 3.92 | 3.92 | 3.93 | 3.93 |  |
|  |  |  |  |  |  |  |  |  |  |  |  |
| **Statistically significant difference** | YES | YES | YES | YES | NO | NO | YES | YES | YES | YES |  |

*Note: The statistically significant difference between compared geographical locations or sampling periods was if F > F crit and p < α (α=0.05); 1^st^ sampling period = February – March 2019; 2^nd^ sampling period = September – October 2019.*

**Table S8:** Results for a Tukey-Kramer´s test (α=0.05) for log10 concentration of PAHs

| **Compared parameter** | ***1^st^ period:* differences between location (1. CB-PR; 2. PR-OS; 3. OS-CB)** | | ***2^nd^ period:* differences between location (1. CB-PR; 2. PR-OS; 3. OS-CB)** | |
| --- | --- | --- | --- | --- |
|  | **Σ20 PAHs** | **BaP** | **Σ20 PAHs** | **BaP** |
| **q value** | 2.89 | 1.62 | 5.43 | 5.40 |
|  | 6.02 | 4.90 | 13.5 | 14.2 |
|  | 6.91 | 4.89 | 3.45 | 4.12 |
| **q-crit** | 3.36 | | 3.36 | |
| **Statistically significant difference** | NO | NO | YES | YES |
|  | YES | YES | YES | YES |
|  | YES | YES | YES | YES |

*Note: The statistically significant difference between compared geographical locations or sampling periods was if q > q crit (α=0.05); 1^st^ sampling period = February – March 2019; 2^nd^ sampling period = September – October 2019; CB = Ceske Budejovice PR = Prague, OS = Ostrava.*

**Table S9** List of abbreviations

| **Abbreviation** | **Full Form** |
| --- | --- |
| ^13^C_12_-DBaiP | ^13^C_12_-dibenzo[*a,i*]pyrene |
| ^13^C_3_-PY | ^13^C_3_-pyrene |
| ^13^C_4_-BaP | ^13^C_4_-benzo[*a*]pyrene |
| ^13^C_6_-5MCH | ^13^C_6_-5-methylchrysene |
| ^13^C_6_-AN | ^13^C_6_-anthracene |
| ^13^C_6_-BaA | ^13^C_6_-benzo[*a*]anthracene |
| ^13^C_6_-BbF | ^13^C_6_-benzo[*b*]fluoranthene |
| ^13^C_6_-BcFL | ^13^C_6_-benzo[*c*]fluorene |
| ^13^C_6_-BghiP | ^13^C_6_-benzo[*g,h,i*]perylene |
| ^13^C_6_-BjF | ^13^C_6_-benzo[*j*]fluoranthene |
| ^13^C_6_-BkF | ^13^C_6_-benzo[*k*]fluoranthene |
| ^13^C_6_-CHR | ^13^C_6_-chrysene |
| ^13^C_6_-CPP | ^13^C_6_-cyclopenta[*c,d*]pyrene |
| ^13^C_6_-DBaeP | ^13^C_6_-dibenzo[*a,e*]pyrene |
| ^13^C_6_-DBahA | ^13^C_6_-dibenzo[*a,h*]anthracene |
| ^13^C_6_-DBahP | ^13^C_6_-dibenzo[*a,h*]pyrene |
| ^13^C_6_-DBalP | ^13^C_6_-dibenzo[*a,l*]pyrene |
| ^13^C_6_-FLA | ^13^C_6_-fluoranthene |
| ^13^C_6_-IP | ^13^C_6_-indeno[1,2,3-*cd*]pyrene |
| ^13^C_6_-PHE | ^13^C_6_-phenanthrene |
| 1-OH-AC | 1-hydroxyacenaphthene |
| 1-OH-NAP | Naphthalene-1-ol |
| 1-OH-PHE | Phenanthrene-1-ol |
| 1-OH-PY | Pyrene-1-ol |
| 2-OH-FL | Fluorene-2-ol |
| 2-OH-NAP | Naphthalene-2-ol |
| 2-OH-PHE | Phenanthrene-2-ol |
| 3-OH-BaA | 3-hydroxybenzo[*a*]anthracene |
| 3-OH-BaP | Benzo[*a*]pyrene-3-ol |
| 3-OH-PHE | Phenanthrene-3-ol |
| 4-OH-PHE | Phenanthrene-4-ol |
| 5MCH | 5-methylchrysene |
| 6-OH-CHRY | Chrysene-6-ol |
| 9-OH-PHE | Phenanthrene-9-ol |
| AC | Acenaphthene |
| ACL | Acenaphthylene |
| AN | Anthracene |
| BaA | Benzo[*a*]anthracene |
| BaP | Benzo[*a*]pyrene |
| BbF | Benzo[*b*]fluoranthene |
| BcFL | Benzo[*c*]fluorene |
| BghiP | Benzo[*g,h,i*]perylene |
| BjFA | Benzo[*j*]fluoranthene |
| BkF | Benzo[*k*]fluoranthene |
| BMI | Body mass index |
| CHR | Chrysene |
| CPP | Cyclopenta[*c,d*]pyrene |
| d_11_-3-OH-BaP | [^2^H]_11_-benzo[*a*]pyrene-3-ol |
| d_7_-1-OH-NAP | [^2^H]_7_-naphtalene-1-ol |
| d_7_-2-OH-NAP | [^2^H]_7_-naphtalene-2-ol |
| d_8_-9-OH-PHE | [^2^H]_8_-phenanthrene-9-ol |
| d_9_-1-OH-PHE | [^2^H]_9_-phenanthrene-1-ol |
| d_9_-1-OH-PY | [^2^H]_9_-pyrene-1-ol |
| d_9_-2-OH-FL | [^2^H]_9_-fluorene-2-ol |
| d_9_-2-OH-PHE | [^2^H]_9_-phenanthrene-2-ol |
| d_9_-3-OH-PHE | [^2^H]_9_-phenanthrene-3-ol |
| DBaeP | Dibenzo[a,e]pyrene |
| DBahA | Dibenzo[*a,h*]anthracene |
| DBahP | Dibenzo[*a,h*]pyrene |
| DBaiP | Dibenzo[*a,i*]pyrene |
| DBalP | Dibenzo[*a,l*]pyrene |
| d-SPE | Dispersive solid phase extraction |
| EI | Electron ionisation |
| ESI | Electrospray ionisation |
| FL | Fluorene |
| FLA | Fluoranthene |
| GC-MS/MS | Gas chromatography coupled with tandem mass spectrometry |
| HAIE | Healthy Aging in Industrial Environment |
| IARC | International Agency for Research on Cancer |
| ICR | Inhalation cancer risk |
| IEM | Institute of Experimental Medicine |
| IP | Indeno[1,2,3-*cd*]pyrene |
| LOQ | Limit of quantification |
| MAX | Maximum |
| MIN | Minimum |
| MRM | Multiple reaction monitoring |
| MS/MS | Tandem mass spectrometry |
| NAP | Naphthalene |
| NIST | National Institute of Standards and Technology |
| OH-PAHs | Monohydroxylated polycyclic aromatic hydrocarbons |
| PAHs | Polycyclic aromatic hydrocarbons |
| PFP | Pentafluorophenyl |
| PHE | Phenanthrene |
| PM | Particulate matter |
| PM2.5 | Particulate phase ≤ 2.5 µm in diameter |
| PY | Pyrene |
| RSD | Repeatability expressed as a relative standard deviation |
| SRM | Standard Reference Material |
| TEQ | Toxicity equivalent concentration |
| TOC | Total organic carbon |
| UHPLC-MS/MS | Ultra-high performance liquid chromatography coupled with tandem mass spectrometry |
| WHO | World Health Organisation |
